# Casting over Metal Method Used in Manufacturing Hybrid Cobalt-Chromium Dental Prosthetic Frameworks Assembles

**DOI:** 10.3390/ma14030539

**Published:** 2021-01-23

**Authors:** Willi Andrei Uriciuc, Horatiu Vermesan, Ancuta Elena Tiuc, Aranka Ilea, Adina Bianca Bosca, Catalin Ovidiu Popa

**Affiliations:** 1 Faculty of Dental Medicine, “Iuliu-Hațieganu” University of Medicine and Pharmacy, 400012 Cluj-Napoca, Romania; willi.uriciuc@umfcluj.ro (W.A.U.); aranka.ilea@umfcluj.ro (A.I.); 2 Faculty of Materials and Environmental Engineering, Technical University of Cluj-Napoca, 400461 Cluj-Napoca, Romania; ancuta.TIUC@imadd.utcluj.ro (A.E.T.); catalin.Popa@stm.utcluj.ro (C.O.P.); 3 Faculty of Medicine, “Iuliu-Hațieganu” University of Medicine and Pharmacy, 400012 Cluj-Napoca, Romania; bianca.bosca@umfcluj.ro

**Keywords:** cobalt–chromium alloy, dental alloys, selective laser melting, accuracy, dental prosthetic framework assemble, casing over metal method

## Abstract

Cobalt–chromium (Co–Cr) alloys are the most widely used materials for removable and fixed dental prosthetic frameworks. The fitting accuracy between these components in dental prosthetic frameworks assembles (DPFAs) is largely influenced by the manufacturing method. This study presents a novel manufacturing method that combined two common techniques for obtaining one single framework: casting of Co–Cr inserts on top of parts previously manufactured by selective laser melting (SLM) of Co–Cr powder (CoM). Horizontal (*n* = 4) and vertical (*n* = 3) surfaces were microscopically analyzed (*n* = 770 count sum). The results revealed a high precision of the process and high fitting accuracy between the hybrid frameworks. The average distance measured between the frameworks in joined position was 41.08 ± 7.56 µm. In conclusion, the manufacturing of Co–Cr alloys DPFA using the CoM method reduced the deformation of hybrid frameworks and improved the joining accuracy between them.

## 1. Introduction

There are a wide variety of designed dental prosthetic frameworks assembles (DPFAs) for the treatment of partial edentation [1]. Most of these dental works are composed of removable partial dentures (RPD) [2] fitted onto dental crowns (DCs) [3], resulting in compound denture (CD) [4].

The dynamic instability of DPFAs’ components during functioning is a common problem causing patient’s discomfort and requires replacement with an improved prosthetic work [1,2,3,4].

The fitting precision of the RPD framework on the milled surface of the primary DC is influenced by the accuracy of the geometrical transfer, which depends on the materials and methods used in the fabrication process [5,6].

The major problem is that, in order to obtain an accurate fitting between the DFPA components, the geometrical pattern of the surface is transferred from the primary to the secondary frameworks using various methods: optical scanning [7], refractory duplication [3,4], or direct resin construction [4]. All these methods use Cobalt–chromium (Co–Cr) alloys [8] (Co:Cr:Mo, Co:Cr:W, Co:Cr:Mo:W) for the fabrication of frameworks through different methods: melting, alloys ingots, and casting in refractory mold (LWT—lost wax technique) [4] or additive manufacturing starting with powder state alloys (SLM—selective laser melting) [9].

Major concerning issues refer to the distortions that occur at the level of secondary frameworks surface during the fabrication process [10].

The accuracy of fitting [11] between assembled frameworks can be affected by the contact of removable partial denture framework with saliva [12] and with the oral cavity soft tissues [13,14]. Wide gaps in this area promote food retention, causing the patient’s discomfort while wearing the prosthetic construction [1,2,3,4].

The LWT method results in gaps ranging between 185 and 352 µm, whereas for the SLM method, these are between 123 and 166 µm [15]. According to [16], clinically acceptable discrepancies are of less than 311 µm, the material influencing also upon this. Several authors reported that the crown margin gap should be smaller than 120 µm for clinical acceptance [17,18,19].

Gurel et al. compared the marginal and internal fit of Co–Cr dental crowns fabricated using two different techniques: CAD/CAM and traditional casting [19].

Comparative studies reported different results regarding the use of the same material [20] for the fabrication of frameworks with various designs, shapes, and lengths [21,22]. The geometric discrepancies of frameworks fabricated using machining process were influenced by the accuracy in sintering contraction of pre-sintered alloys [23]. The same problem appeared in the sintering process of SLM manufactured frameworks.

Literature data reported that the LWT method and CAD-CAM technology have statistically similar results. Moreover, the axial discrepancy values of the SLM technique (70 ± 19 µm) were significantly higher than those of LWT (45 ± 16 µm) [24]. No clear information could be found in literature about the superiority of CAD-CAM technology over the casting technique regarding the marginal adaptation of dental frameworks [25].

Other studies showed that the marginal fit values of the Co–Cr alloys frameworks greatly depended on the fabrication methods and, occasionally, the alloy systems [26].

The present study aims to propose a way to improve the accuracy under good reproducibility conditions for the connection between the components (DCs frameworks and RPD framework) of dental prosthetic assembles (CD), and to disseminate the step by step technological process related to the new manufacturing methods, in order to complete the data published in the literature.

The innovative method presented in this study optimized the fitting accuracy between frameworks and between the soft oral tissues and the prosthetics frameworks [27], resulting in hybrid dental prosthetic frameworks assemblies.

## 2. Materials and Methods

In the present study, a Co–Cr dental prosthetic frameworks assemble was made using the combination of two manufacturing methods: SLM by powder technology and casting by LWT. The combination of the two technologies was achieved by using a SLM framework as an insert piece in the refractory mold and casting over a LWT framework that was named hybrid framework. A hybrid framework was obtained from a casted piece reinforced with a metallic piece.

For this study, a carbon–steel master cast was used (C1). The cast consisted of a cone trunk (h = 4 mm, d = 9 mm) with cylindrical base (h = 3 mm, d = 12 mm), which idealized a clinical dental abutment (the dental–periodontal support) and a circular platform (h = 1 mm, d = 29 mm) that was screwed to the abutment. The platform idealized the clinical gingival tissue surrounding the tooth (the gingival–bone support).

The master cast was designed to receive the Co–Cr assembled structures, which exhibited a fixed and a detachable component of compound dentures (CD).

**Figure 1:**
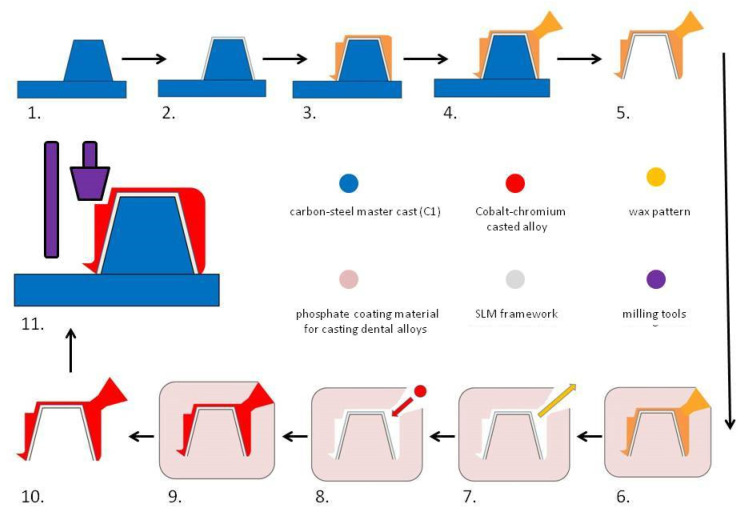
Diagram showing the technological process of manufacturing the dental crown hybrid framework: (1) master cast (blue); (2) Co–Cr coping produced by SLM (grey); (3) wax pattern (orange) milled by CNC machine fitted over metal coping; (4) spruing wax (orange); (5) wax pattern over the metal coping detached from the master cast; (6) coating in refractory material (pink); (7) lost wax technique and the resulting mold (white); (8) casting over metal coping (grey) molten alloy (red); (9) cooling of the alloy in the mold; (10) dental crown hybrid framework (red-grey); and (11) mechanical preparation of the parallel surface using milling tools (purple).

**Figure 2:**
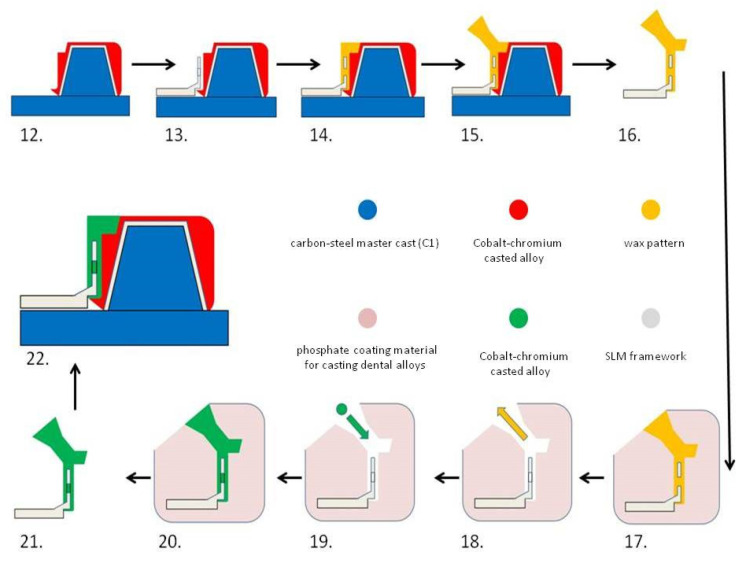
Diagram showing the technological process of manufacturing the removable partial denture hybrid framework: (12) dental crown hybrid framework (red–grey) on the master cast (blue); (13) Co–Cr framework produced by SLM (grey); (14) wax pattern (orange) produced by manual additive method (orange) joining Co–Cr framework and dental crown hybrid framework external surface; (15) spruing wax (orange); (16) wax pattern over metal coping detached from the master cast; (17) coating in refractory material (pink); (18) lost wax technique and the resulting mold (white); (19) Casting over metal framework (grey) and the molten alloy (green); (20) cooling of the alloy in the mold; (21) removable partial denture hybrid framework (green–grey); (22) removable partial denture hybrid framework fitted on the DC hybrid framework and the resulting hybrid dental prosthetic frameworks assembles.

For a better understanding of the technological process, the fabrication of hybrid dental prosthetic frameworks assembles was presented in two detailed diagrams (Figure 1 and Figure 2).

**Figure 3:**
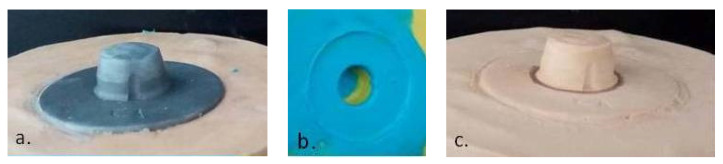
(**a**) Steel master cast (C1); (**b**) simulation of shape transfer from the prosthetic field (steel master cast) using a silicon impression; (**c**) dental laboratory stone cast.

The simulation of data (shape, volume) transfer from the clinical prosthetic field (steel master cast) (Figure 3a) to the dental laboratory stone cast (Figure 3c) was performed by an analog method, using A-silicon impression (Figure 3b). One-step impression was made with two materials consisting of vinyl-poly-siloxane, putty (yellow—Figure 3b), and light body (blue—Figure 3b) (Elite P & P-hydrophilic—Zhermack, Italy) supported in a metal tray similar to the clinical procedure in the oral cavity.

**Figure 4:**
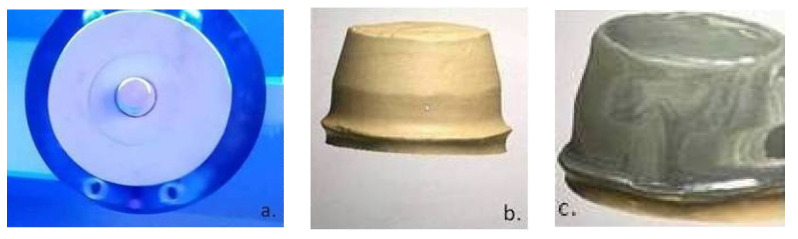
(**a**) Scanning of the dental laboratory stone cast using structural blue light; (**b**) computer-aided design (CAD) software screen view of a digitalized dental cast (abutment); (**c**) CAD software screen view of the 3D coping framework design.

For the digitization of the abutment geometry, a dental laboratory scanner with blue light scanning technology (MEDIT-T, Medit Corp. Seoul, Korea) was used, and the information was transformed into STL format (Figure 4a). The file was opened in a Software computer-aided design (CAD) (3Shape, Copenhagen, Denmark) (Figure 4b) to obtain a copy of the framework designing processing (Figure 4c).

**Figure 5:**
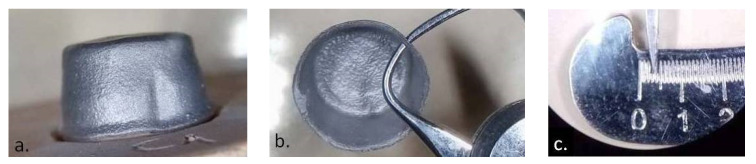
(**a**) CAD Software screen view of the 3D copy framework design; (**b**) external surface of Co–Cr copy framework; (**c**) the thickness of the copy wall measured with a mechanical micrometer; (**d**) the scale of the instrument indicated a thickness of 35 µm.

Using a professional 3D printer (MYSINT 100—Sisma SPA, Arezzo, Italy), a copy framework (Figure 5) was made from Co–Cr powder with 10–40 µm particle size and round/spherical shape [22] (Co:Cr:Mo:W=63.9:24.7:5:5.4 in wt%; Mediloy S-Co-Bego, Bremen, Germany) using SLM technology.

**Figure 6:**
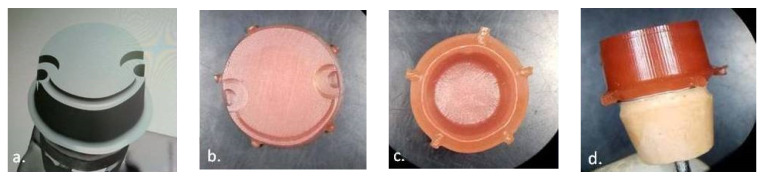
(**a**) Digital design of the complementary dental crown framework with geometrical surface; wax pattern milled by the five-axes CNC machine; (**b**) external view; (**c**) internal view; (**d**) para-axial view (pattern on the dental laboratory stone cast abutment).

The external surface of the copy framework was scanned using blue light scanning technology (MEDIT-T, Medit Corp. Seoul, Korea) and the information was transformed into STL format. The file was opened in a CAD Software (3Shape-Denmark) in order to design the digital shape of the complementary framework (Figure 6a). The 3D design information in STL format was sent to computer-aided manufactured (CAM) production. A five-axes CNC machine (Corintec 650I—Imes-Icore Gmbh, Eiterfeld, Germany) was used for processing the wax blocks and for milling the patterns for the complementary dental crown framework (Figure 6b,c).

**Figure 7:**
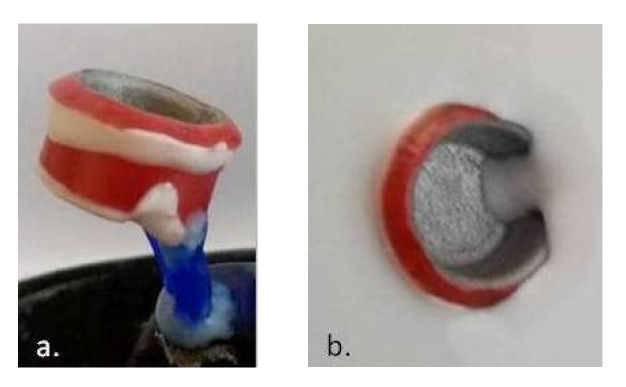
(**a**) Preparation of the wax pattern for molding; (**b**) insertion of the metallic framework and the wax pattern during the process of molding.

The wax pattern was used for the fabrication of the refractory mold (Figure 7). In advance, the external surface of Co–Cr insert framework was machined (speed = 15 × 10^3^ rpm) using a dental laboratory micro motor (Marathon Multi 600—Saeyang, Daegu, Korea) and sand-blasted with aluminum oxide (175 µm granulation) at 3 atm pressure, using a sand blasting machine (Easyblast blaster-Bego, Bremen, Germany).

The Co–Cr alloy (Co:Cr:W:Fe = 55.2:24:15:4 in wt%; Heraenium PW-Kulzer, Germany) was casted into refractory mold, over the copy of the metallic insert using an induction melting casting machine (model T-Fornax-Bego, Bremen, Germany).

**Figure 8:**
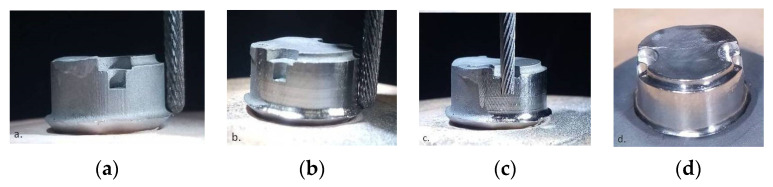
(**a**,**b**) Milling the lower step; (**c**) milling the pin-lock; (**d**) dental crown framework.

The design of the surface geometry was very important, and it was described in the horizontal plane as a lower step (Figure 8a,b) and an upper shoulder that reproduced the shape “Ω” (Figure 8d) and ensured the vertical rest for the removable partial denture framework. The para-axial geometry was designed as a vertical wall (0° angle) (Figure 8a, b) that also followed the shape “Ω”, ensuring the retention of the removable partial denture framework on the surface of the dental crown framework. The pin-locks (Figure 8c), designed at the end of the omega shape, allowed for the simultaneous path of insertion and detachment. The vertical wall that followed the shape of the shoulder was projected at an angle of 3° wider than the vertical wall, which followed the shape of the step.

The Co–Cr dental crown metallic hybrid framework was mechanically machined in a parallel milling machine (AF-300—Amann Girrbach, Koblach, Austria) and polished with mirror texture (Figure 8d)

The retentive geometry of milled surface was tested though the construction of self-hardening resin pattern (Pattern Resin—GC Company, Tokyo, Japan) for RPD framework with the possibility to detach the pattern after solidification.

The geometry of the mounting surface between the frameworks was transferred from one to the other by direct pattern and casted by Co–Cr alloy in refractory mold over the insertion of the metallic removable partial denture framework.

**Figure 9:**
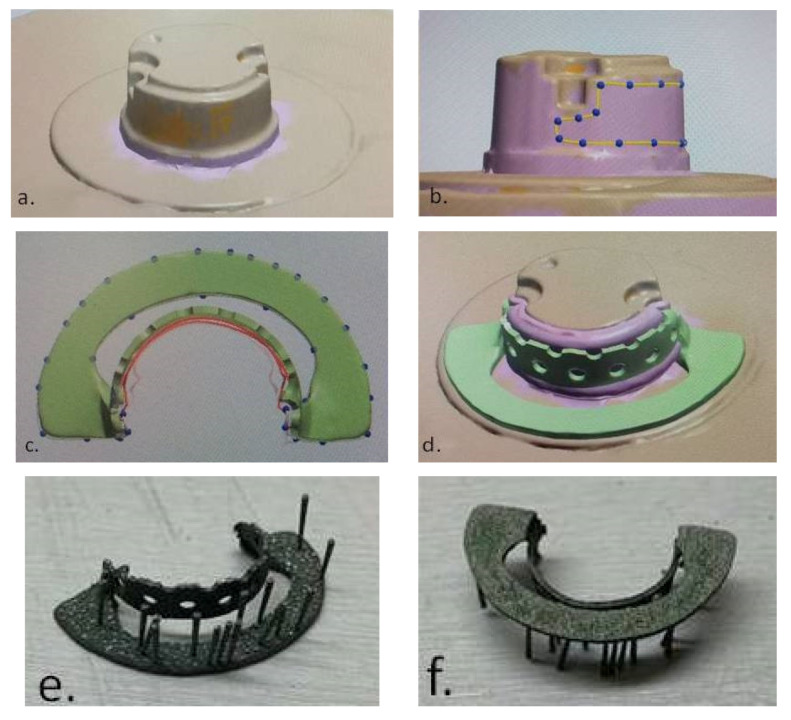
(**a**) Dental crown framework (STL format); (**b**) work in progress design; (**c**) virtual design of removable partial denture framework; (**d**) removable partial denture framework design in relation with dental crown framework; (**e**) removable partial denture framework (external surface); (**f**) removable partial denture framework (mucosal surface).

The metallic RPD framework was made of the same Co–Cr powder that was used for the SLM technology after the DC framework surface was scanned and removable partial denture virtual framework design was made (Figure 9).

The RPD framework design was performed at some distance, and retention holes were made in the DC–RPD junction area (Figure 9d). The fitting between frameworks was obtained by casting over the metal with a removable partial denture framework as a metal insert. Casting procedure was made in refractory mold, using the same alloy as for the dental crown hybrid framework (Figure 2).

**Figure 10:**
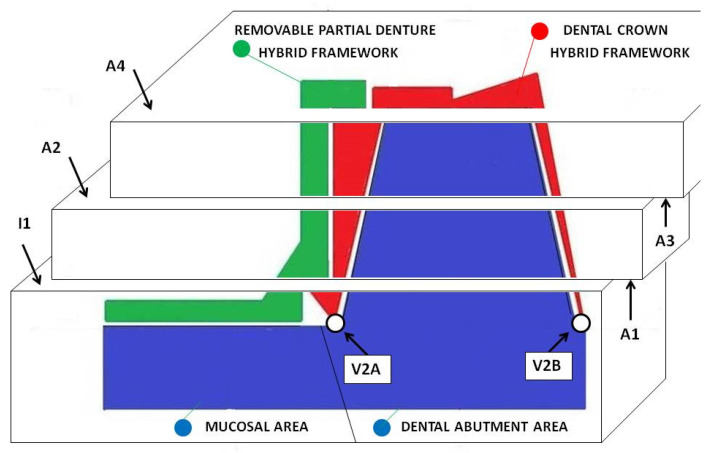
Geometric map of sections through frameworks assemble (removable partial denture hybrid framework—green, dental crown hybrid framework—red) in contact with master cast (dental abutment and mucosal area—blue); I1, A1, A2, A3, and A4 are the areas resulting from horizontal sections through the assemble; V2A and V2B are the areas resulting from vertical sections at the marginal–cervical area of dental crown hybrid framework at the level of contact with the dental abutment.

Prior to the initial coupling, the first horizontal section was made to create access for measuring the gaps between the frameworks at the level of the initial area I1 (Figure 10).

After the initial coupling, the joint surfaces between the two hybrid frameworks of the dental prosthetic frameworks assembles were mechanically machined to obtain a mirror-like texture, following the cutting of the other sections to obtain the areas A2, A3, A4, and to finish the area A1.

The following horizontal sections were made: one at the supra-cervical level (resulting the surfaces I1 and A1), one at the middle level (resulting the surfaces A2 and A3), and one straightening the occlusal (upper) surface (resulting the surface A4); between all sections, there were 2 mm in vertical plane.

**Figure 11:**
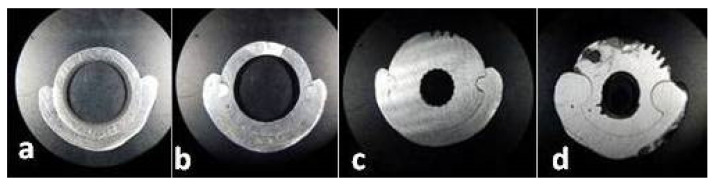
Sections through the framework assemble: (**a**) A1; (**b**) A2; (**c**) A3; (**d**) A4.

All sections (Figure 10) were made by rotary cutting using a 0.5 mm silicon carbide (SiC) disc at a speed of 15,000 rpm and then finished with sandpaper (500, 1200) (Figure 11).

The frameworks: dental crown hybrid framework (red—hybrid dental crown framework) and removable partial denture hybrid framework (green—hybrid removable partial denture framework) joined in prosthetic assemble and placed on prosthetic field (blue) were analyzed in horizontal and vertical sections (Figure 10). Using the specific software (Image Analysis Software, Olympus Europa SE& Co KG, Hamburg, Germany) attached to the stereo microscope (GX51, Olympus Europa SE& Co KG Hamburg, Germany), micrographic segments were captured and the gaps were measured. The measurements were made on micrographic segments at 200× magnification.

A total of 500 measured lengths were analyzed, five lengths for each of the 100 microscopic segments and four areas of horizontal sections (A1, A2, A3, and A4), (Figure 10 and Figure 11).

**Figure 12:**
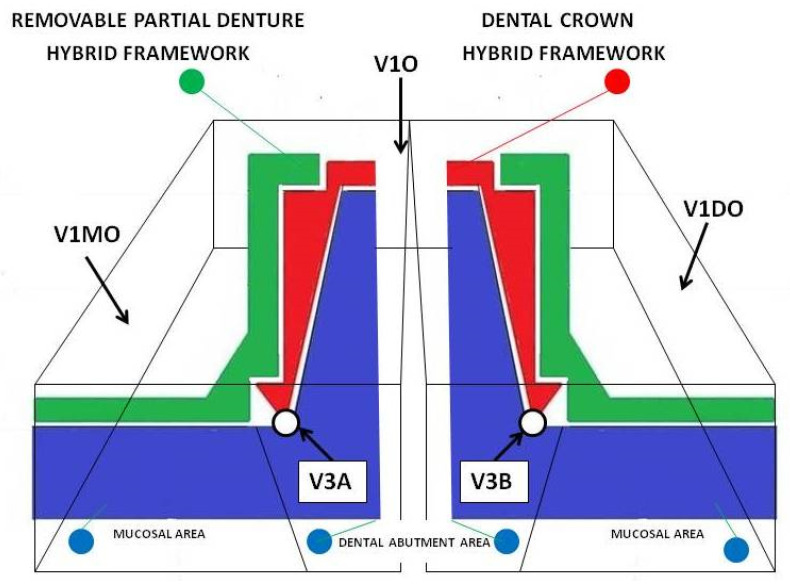
The areas V3A and V3B resulted from the vertical sections at the marginal–cervical zone of the dental crown hybrid framework at the level of contact with the dental abutment; V1MO, V1O, and V1DO are the areas that result from vertical section true removable partial denture framework at the level of contact with mucosal area.

At the same time, the micrographs on the V1 surfaces (mesial—M, oral—O, and distal—D) were measured, which represented the accuracy of joining of the removable partial denture hybrid framework to the mucosal area of the master cast (Figure 12).

**Figure 13:**
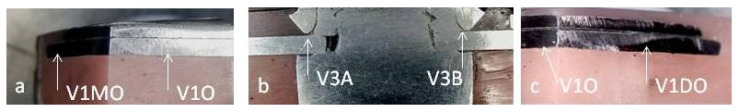
Sections through the framework assemble: (**a**) V1MO, V1O; (**b**) V3A, V3B; (**c**) V1O, V1DO.

Moreover, the micrographs resulting from V2 and V3 sections: V2A, V2B (Figure 10), V3A, and V3B (Figure 12 and Figure 13) (A and B are the areas of the resulting surfaces) were measured, which represented the accuracy of the combination between the dental crown hybrid framework and the dental abutment in the marginal–cervical area.

## 3. Results

The results indicated a total average of 41.08 µm for the fitting accuracy between the dental prosthetic assemble frameworks, with a minimum measured average of 32.88 µm and a maximum of 53.02 µm with standard deviation per measured segment of 7.56 µm (Table 1).

**Table 1:** The measurements (µm) between dental crown–framework and removable partial denture framework at joining levels at four areas (A) of horizontal section through the dental prosthetic detachable assemble (Figure 14).

A	Mean (µm)	Minimum Mean (µm)	Maximum Mean (µm)	Standard Deviation Mean (µm)	Count Sum (Length)
A1	48.34	43.48	54.06	4.00	95
A2	37.63	28.99	55.51	10.19	135
A3	31.89	20.14	46.24	9.98	135
A4	46.49	38.94	56.28	6.08	135
A	41.08	32.88	53.02	7.56	500

The purpose of the analysis at the level of the first section before and after the initial decoupling of the dental prosthetic frameworks assembles frameworks was to compare the distance between the two pieces at the time of the fabrication (I) and at the time of functioning (A).

**Figure 15:**
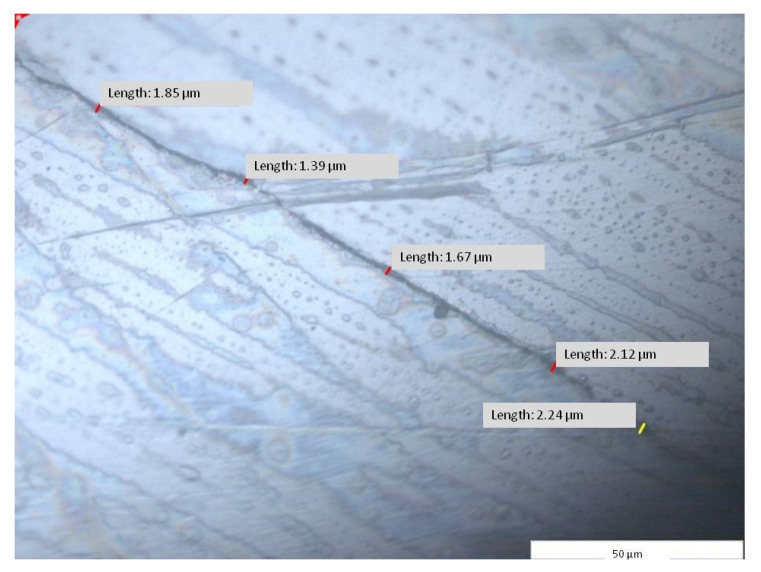
Micrographic segments (1000×) after measuring the length of the gap between the joined frameworks of the dental prosthetic frameworks assembles (I1—area 1, segment g2).

**Figure 16:**
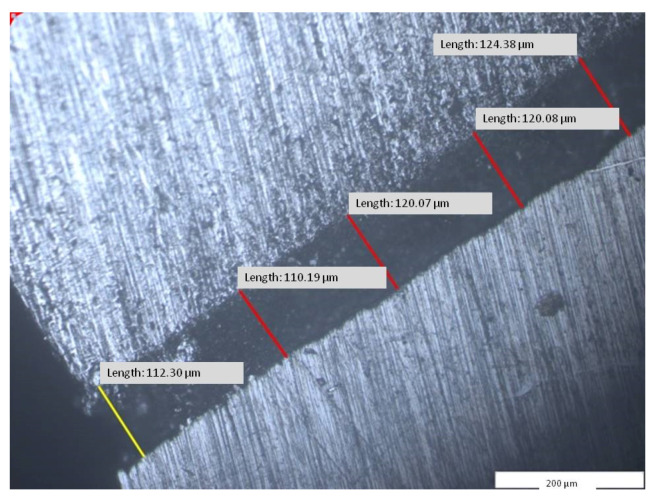
Micrographic segment (200×) after measuring the length of the gaps between dental abutment and dental crown framework at cervical area (V2B—area, segment g2).

The results led to a difference between I1 (Figure 15) and A1 (ΔAI = 39.98 µm) for the average of the measurements with a differences at the minimum, maximum, and the standard deviation (Table 2 and Figure 16).

The analysis of the marginal closure of the dental crown hybrid framework (Figure 10—red) with the prosthetic abutment (Figure 10—blue) at the marginal cervical level was analyzed on micrographs taken from four surfaces (V2A and V2B, V3A and V3B). The result was the total mean (V23) between distances measured at the level of all four surfaces and presented in Table 3.

The results of the 55 measured distances was an average of 112.15 µm, with a minimum of 92.72 µm, a maximum of 138.74 µm, and a standard deviation per segment of 16.36 µm (Figure 16).

**Figure 17:**
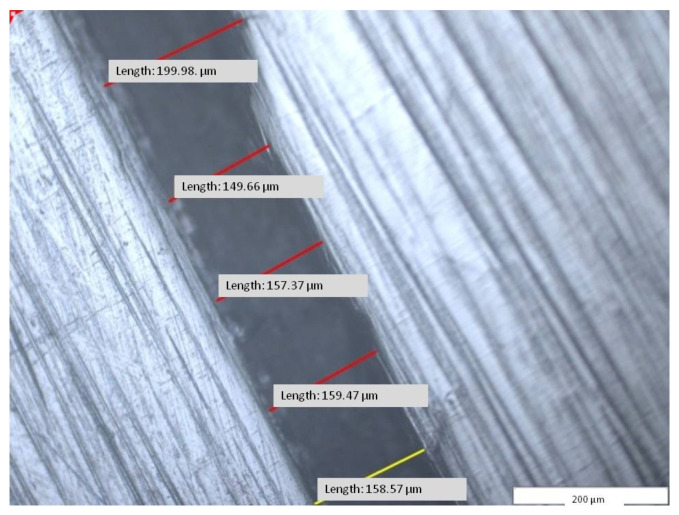
Micrographic segment (200×) after measuring the length of the gap between removable partial denture framework and mucosal area at the V1 area (V1—area, segment d6).

A number of 215 distances were measured between the removable partial denture framework and the mucosal surface, found on the three areas (A) resulting from V1 section: V1MO—mesial–oral area, V1O—oral area, and V1DO—distal–oral area. The total average (V1) of the measurements was 157.53 µm, with a minimum value of 137.24 µm, maximum value of 176.69 µm, and a standard deviation per segment of 14.28 µm (Table 4 and Figure 17).

The V-average distance measured at surfaces V1, V2, and V3 (Table 5 and Figure 17) was found between the master cast and the hybrid frameworks on the parts that were produced by CAD and CAM-SLM technology, without their surface being mechanically machined by cutting, only sandblasted with aluminum oxide.

The A—average distances were measured at surfaces A1, A2, A3, and A4 (Table 1) between the dental crown hybrid-framework and the removable partial denture hybrid framework on the parts that were produced by casting over metal technology (CoM).

All the patterns made for casting in refractory mold were also produced by CAD and machined by milling in wax blocks using a computer numerical control machine (CNC). The distances difference (ΔVA) measured between frameworks produced by CoM- casting over metal method and SLM are shown in Table 6.

## 4. Discussion

The final goal of the present study was to propose a novel manufacturing route that ensures a high fitting precision between the components of hybrid dental prosthetic frameworks assemblies. In order to assess the obtained results in view of validation leading to clinical application, the existing reports of other results in recent specialized literature were considered.

The classic assemble of frameworks (dental crown and removable partial denture) is manufactured as two separate elements, consisting of the dental crown (with fitting surface in oral area), which will be cemented on the prepared abutment tooth, and the removable partial denture (with fitting surface in mucosal area), which will have the possibility to be removed and refit in the initial position.

One of the problems is the poor adaptation of the internal and marginal surface of a piece to the shape it was intended to copy in manufacturing process.

For dental crowns, a larger gap between the inner surface and the prosthetic abutment, even if it will generate a higher degree of free movement between the two parts, will not affect the maintenance, since the dental crown is fixed by screwing or cementing on the prosthetic field. The marginal misfit of a dental crown can produce gingival lesions and medical problems.

The fitting between frameworks must be improved even if the framework of dental crown will be cemented or screwed to prosthetic abutment. In the cementation method for fixing the dental crown, the large internal gap will be filled with a thick layer of cement, and will eventually crack, because the cement has good resistance to fracture only in a thin film. In the screwing method for fixing the dental crown on dental implant abutment, the masticatory forces will unscrew the framework.

Other studies showed the marginal and internal connection between the dental or implant prosthetic abutment and the internal structure of a dental crown.

Several studies focused on the connection between the dental crown framework and the prosthetic abutment (the natural tooth) [19] or the implant abutment [23,28].

In these cases, the two frameworks involved will be clinically fixed in the oral cavity, by cementing or screwing, and will not be functional parts that require continuous coupling and detachment in order to be daily cleaned, as the dental frameworks prosthetic assemble.

Ruscheli et al. reported an average value of 195.2 ± 14.2 µm for the internal and marginal fitting between the dental implant abutment and the metal coping [28].

Hang-NgaMai et al. reported internal discrepancy of fitting between implants abutments and milled Co–Cr framework ranging between 132.3 ± 70.8 and 148.8 ± 50.7 µm [23].

In other studies, the analyzed frameworks had clinically acceptable discrepancies (<311 µm) and the material influenced the fitting accuracy [16]. Several authors reported that the crown marginal gap should not exceed 120 µm for clinical acceptance [17,18,19]. Comparative studies showed different results for the same material used for the manufacturing of different frameworks in terms of design, shapes, and size [20,21].

Our results indicated a better average value (V23 = 112.15 ± 16.36 µm) and a fitting accuracy ranging between 92.72 and 138.64 µm. The results are close to those of Gurel et al., who reported gap measurements of 113.36 ± 19.10 µm [19].

Other studies focused on the precision of joining metal structures that make up a telescopic system (double crown attachment); these are functional frameworks that will allow the coupling and detachment of the metal parts.

In these cases, only the primary framework is cobalt–chromium manufactured using lost wax method or selective laser melting method. The secondary framework that joins with precision at the surface of primary framework is gold manufactured by electrochemical deposition method directly on the surface of primary framework. These type of functional frameworks assembles have higher costs of manufacturing process because of the high price of both the gold electrolytic liquid and the device.

For both cases, the shape of the connection between metal framework is conical (internal surface of dental crown with prosthetic abutment) or cylindrical (double crown attachment).

Regarding the removable partial denture framework, numerous studies analyzed the precision between the metallic framework and mucosal ridge or natural tooth [10,11,12,14,15,16,17,21].

Stern et al. reported differences ranging between 123 and 166 µm regarding the fitting precision of removable partial denture framework manufactured through the SLM method [15].

Tasaka et al. discussed removable partial denture frameworks with extra-coronal clasps, manufactured using selective laser sintering method [16].

The frameworks in the present research article were made to fit the prosthetic field that contends the teeth remaining in the oral cavity and the gingival tissue in the edentulous areas.

The shapes of the prosthetic field are not geometrical and the clinical acceptable discrepancies are correct.

In the case of dental crown frameworks that must be joined in mechanically detachable assembles with a removable partial denture framework, both being metallic pieces, the joining accuracy must be optimal.

The functional fitting between the non-retentive surfaces of the geometrical metallic pieces (cones or cylinders) must be under 50 microns, because even with a 120 microns value of the gap, the fitting would lack freedom and would need complementary retention (special semi-precision dental attachments).

The present study showed that the total average value for V1 was 157.53 ± 14.28 µm and the fitting accuracy between removable partial denture hybrid framework and the mucosal ridge area ranged between 137.24 and 176.69 µm of the master cast. These data are consistent with the results of other studies.

The joining accuracy between both frameworks provides protection and support for the removable partial denture against dislocations forces. At the same time, the dimension of gaps between framework surfaces determinates the guidance of removable partial dentures insertion on the dental crown.

In a prosthetic assemble, the fitting is achieved between two metal parts, the implant abutment and the framework, but the results can be used as references for analyzing the fitting of two frameworks; just the manufacturing method has to be taken into account.

At present, there are no available data regarding the accuracy of the connection between the dental crown framework and the removable partial denture framework, only between each of these frameworks and the prosthetic field.

The results regarding the fitting between the dental crown hybrid framework and the removable partial denture hybrid framework produced by the here proposed CoM- casting over metal method (A = 41.08 ± 7.76 µm and a range between 3288 µm and 53.02 µm) are much better than other results obtained by using other methods.

The lost wax method and the casting of chromium–cobalt alloys in the mold produce frameworks that can only be joined by adaptation using mechanical processing by cutting. The adaptation process is time consuming, and the results are never as expected, the join being either with friction or freedom of movement.

None of these types of joints are suitable for the operation and wearing the removable partial dentures in combination with a dental crown.

The selective laser melting method using cobalt–chromium powder produces, in a time-effective manner, structures whose joining requires adaptation, if this has not been already done, with a degree of freedom between the structures.

The casting over metal method proposes the combination of the two manufacturing methods for obtaining a single framework. The joint between the fixed framework (dental crown) and the removable framework (removable partial denture) is made by casting cobalt–chromium alloy in the mold, over the selective laser melting metallic framework that has already been manufactured.

The V value is the average of the measurements obtained from the joint level between the framework components that were manufactured by SLM method and the prosthetic field:

- V1 is the value of the average obtained from the measurements at the level of fitting between the removable partial denture framework and the mucosal area.
- V23 is the value of the average obtained from the measurements at the level of fitting between the dental crown framework and the prosthetic abutment.

V = (V1 + V23)/2

V = (157.53 +112.15)/2

V = 269.68/2

V = 134.84 (µm)

The value A is the average of the measurements obtained from the joint level between the two frameworks components that were manufactured by casting over metal method (A = 41.08 µm).

The value of ΔVA is the difference between V value and A value.

ΔVA = V − A

ΔVA = 134.84 − 41.08

ΔVA = 93.76 (µm)

A = V − ΔVA

A = 134.84 − 93.76

A = 41.08 (µm)

The value of ΔVA = 93.76 (µm) represents the difference between selective laser melting method and casting over metal method apply in oral cavity.

The value of A (A = 41.08 µm) represents approx. 30% from value of V (V = 134.84 µm), and the difference ΔVA (ΔVA = 93.76 µm) represents approx. 70%.

## 5. Conclusions

Based on the results of this study, the following conclusions were drawn:

1. Using the casting over metal method, the fitting between the removable partial denture hybrid framework and the dental crown hybrid framework had better value than the fitting between those frameworks and their selective laser melting components in the proximity of the prosthetic field.
2. CoM, the casting over metal method, represents an innovation in the fabrication process using cobalt–chromium alloys, combining CAD/CAM technology with the classic casting technology and resulting in high precision dental prosthetic frameworks assembles.
3. The clinical consideration after 20 years of applying dental prosthetics manufacturing, corroborated with this 70% improvement of fitting, is that the patient’s comfort during wearing the combined denture (dental crown and removable partial denture) is improved, and the results are maintained for a longer time.

## Figures and Tables

**Figure 14:**
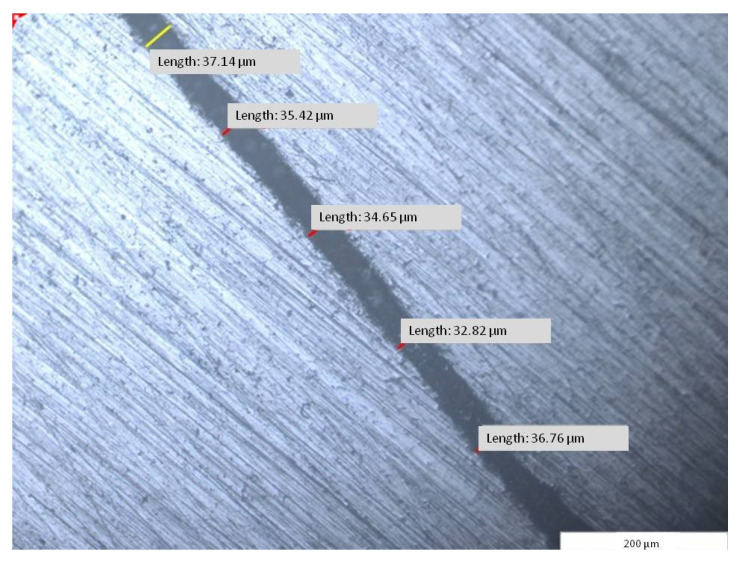
Micrographic segment (200×) after measuring the length of the gap between the joined frameworks of the dental prosthetic frameworks assembles (A3—area 3, segment g7).

**Table 2:** The measurements (µm) of distance between dental crown framework and removable partial denture framework at joining levels for two areas (A): area 1, at the time of fabrication (I1) and at the time of functioning (A1) of dental prosthetic frameworks assembles.

A	Mean (µm)	Minimum Mean (µm)	Maximum Mean (µm)	Standard Deviation Mean (µm)	Count Sum (Length)
A1	48.34	43.48	54.06	4.00	95
I1	8.36	5.93	11.55	1.99	210
ΔAI	39.98	37.35	42.51	2.01	-

**Table 3:** The measurements (µm) between dental crown framework and dental abutment at the four cervical areas (A).

A	Mean (µm)	Minimum Mean (µm)	Maximum Mean (µm)	Standard Deviation Mean (µm)	Count Sum (Length)
V2A	58.78	44.79	70.24	8.78	15
V2B	160.18	127.43	208.80	29.45	15
V3A	93.68	81.86	104.19	7.85	10
V3B	135.96	116.82	171.36	19.37	15
V23	112.15	92.72	138.64	16.36	55

**Table 4:** The measurements of distances (µm) between removable partial denture framework and mucosal area.

A	Mean (µm)	Minimum Mean (µm)	Maximum Mean (µm)	Standard Deviation Mean (µm)	Count Sum (Length)
V1MO	188.69	168.99	205.89	13.72	75
V1O	118.87	94.18	142.10	17.26	85
V1DO	165.04	148.56	182.09	11.87	55
V1	157.53	137.24	176.69	14.28	215

**Table 5:** The measurements of the distances (µm) between frameworks and master cast.

A	Mean (µm)	Minimum Mean (µm)	Maximum Mean (µm)	Standard Deviation Mean (µm)	Count Sum (Length)
V1	157.53	137.24	176.69	14.28	215
V23	112.15	92.72	138.64	16.36	55
V	134.84	114.98	157.52	15.32	270

**Table 6:** The distance difference ΔVA (µm) between frameworks produced by CAD/CAM and CoM method.

A	Mean (µm)	Minimum Mean (µm)	Maximum Mean (µm)	Standard Deviation Mean (µm)	Count Sum (Length)
V	134.84	114.98	157.52	15.32	270
A	41.08	32.88	53.02	7.56	500
ΔVA	93.76	82.10	104.50	7.76	-

## Data Availability

Data sharing is not applicable to this article.
